# Mitochondrial transplantation for osteoarthritis: from molecular mechanisms to clinical translation

**DOI:** 10.3389/fimmu.2026.1716906

**Published:** 2026-03-23

**Authors:** Ying Liu, Yang Liu, Na Zhang, Haizhuan An, Liangyu Mi, Yanan Gao, Ke Xu

**Affiliations:** 1 Department of Rheumatology and Immunology, Shanxi Bethune Hospital, Shanxi Academy of Medical Sciences, Third Hospital of Shanxi Medical University, Tongji Shanxi Hospital, Taiyuan, China; 2 Third Hospital of Shanxi Medical University, Shanxi Bethune Hospital, Shanxi Academy of Medical Sciences, Tongji Shanxi Hospital, Taiyuan, China

**Keywords:** extracellular vesicles, mitochondrial dysfunction, mitochondrial transplantation, mitophagy, osteoarthritis, oxidative phosphorylation, regenerative medicine, stem cells

## Abstract

Osteoarthritis (OA) is the most prevalent chronic degenerative joint disorder worldwide, characterized by progressive cartilage degradation, subchondral bone remodeling, synovial inflammation, and impaired mobility. Growing evidence has established mitochondrial dysfunction—including impaired oxidative phosphorylation (OXPHOS), excessive reactive oxygen species (ROS) generation, disrupted mitochondrial dynamics, and dysregulated mitophagy—as an early and pivotal driver of OA pathogenesis. These bioenergetic failures not only disrupt chondrocyte metabolism but also amplify inflammation, matrix degradation, and cell death. In recent years, mitochondrial transplantation has emerged as a revolutionary therapeutic paradigm, aiming to restore cellular homeostasis by delivering functional mitochondria into damaged chondrocytes. This review systematically summarizes the molecular mechanisms of mitochondrial dysfunction in OA and highlights three major therapeutic strategies: (1) cell-based approaches, particularly mesenchymal stem cell (MSC)-mediated mitochondrial transfer via tunneling nanotubes (TNTs) or extracellular vesicles (EVs); (2) cell-free approaches, utilizing purified mitochondria or MitoEVs for direct transplantation; and (3) engineered mitochondrial transplantation, integrating bioengineering, nanotechnology, and genetic modification to enhance mitochondrial quality, delivery efficiency, and therapeutic persistence. We further discuss opportunities and challenges in clinical translation, including standardization of mitochondrial preparation, optimization of delivery systems, immunological safety, and regulatory classification. Collectively, mitochondrial transplantation represents a disruptive strategy that directly addresses the bioenergetic collapse of chondrocytes and offers a promising avenue for disease-modifying therapy in OA. Future advances in mechanistic elucidation, technological optimization, and multicenter clinical trials will be crucial to transform “mitochondrial medicine” from experimental concept to clinical reality.

## 1 Introduction

Osteoarthritis (OA) is a prevalent chronic degenerative joint disorder characterized by progressive cartilage degradation, subchondral bone remodeling, osteophyte formation, synovial inflammation, and impaired joint mobility (1–3). According to epidemiological surveys, the global prevalence of OA increased from 247.51 million to 527 million between 1990 and 2019, representing a 13.25% rise, with approximately 25% of individuals over the age of 50 experiencing symptomatic OA in the hands, hips, or knees (4–6).

Although osteoarthritis (OA) is widely recognized as a whole-joint disease involving pathological alterations across multiple joint tissues—including subchondral bone remodeling, osteophyte formation, synovial inflammation, infrapatellar fat pad dysfunction, and meniscal degeneration—progressive cartilage degeneration remains the central pathological hallmark most closely associated with disease progression and functional impairment (7). Increasing evidence indicates that other joint tissues actively contribute to OA pathogenesis, particularly in knee OA, where biomechanical and biochemical changes in the infrapatellar fat pad and degenerative alterations of the meniscus amplify inflammatory signaling and tissue crosstalk within the joint microenvironment (8–10).

Articular cartilage is an avascular tissue whose extracellular matrix (ECM) homeostasis relies on metabolically flexible chondrocytes that adapt to fluctuating mechanical and nutritional signals. While glycolysis accounts for approximately 75% of ATP generation under physiological conditions, mitochondrial oxidative phosphorylation (OXPHOS) not only contributes the remaining 25% but is also essential for sustaining the mitochondrial membrane potential (ΔΨm) required for anabolic processes and Ca²^+^-dependent mechanotransduction (11). In OA, mitochondrial dysfunction primarily manifests as respiratory chain impairment rather than a reduction in mitochondrial number (12).

A growing body of evidence indicates that mitochondrial dysfunction—including impaired OXPHOS, excessive reactive oxygen species (ROS) production, and mitochondrial DNA (mtDNA) damage—represents an early and pivotal driver of OA pathogenesis rather than a mere bystander (13, 14). Advances in single-cell transcriptomics have further revealed heterogeneity among chondrocyte subpopulations, such as inflammatory and prehypertrophic phenotypes, each exhibiting distinct metabolic signatures closely linked to mitochondrial status (15).

Despite extensive attempts to use antioxidants or metabolic modulators to restore mitochondrial function, clinical translation has been hampered by limited efficacy, insufficient targeting, and potential adverse effects (16, 17). Against this background, mitochondrial transplantation—a strategy involving the direct delivery of intact, functional exogenous mitochondria into damaged cells or tissues—has emerged as a revolutionary organelle replacement therapy. This concept was first validated in myocardial ischemia–reperfusion injury models (18) and is supported by the discovery of natural intercellular mitochondrial transfer phenomena (19). More importantly, recent animal evidence suggests that mitochondrial transplantation may delay cartilage degeneration and partially restore metabolic homeostasis, indicating its potential as a disease-modifying strategy for OA (20).

Nevertheless, research on mitochondrial transplantation in OA remains in its infancy. Fundamental questions regarding its precise cellular targets, molecular mechanisms, optimal delivery strategies, and long-term safety have yet to be fully addressed. This review aims to provide a comprehensive overview of the role of mitochondrial dysfunction in OA pathogenesis, discuss therapeutic strategies involving cell-dependent, cell-free, and engineered mitochondrial transplantation, and offer perspectives on the clinical translation of “mitochondrial medicine.”

## 2 Mitochondrial dysfunction

### 2.1 The “dual-engine” imbalance of chondrocytes: dysregulated coupling between OXPHOS and glycolysis

**Figure 1:**
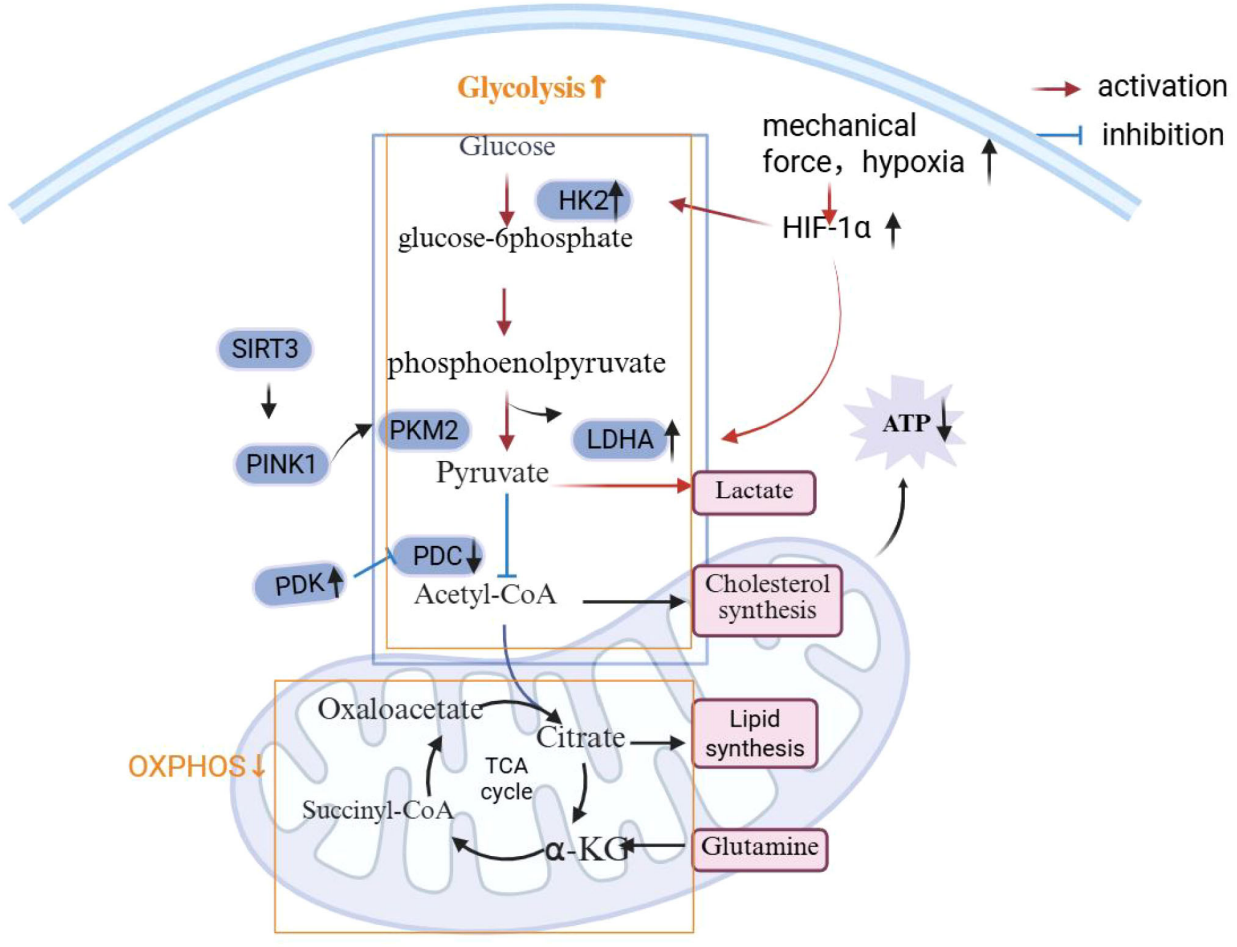
Dual-engine imbalance in osteoarthritic chondrocytes. Under physiological conditions, chondrocytes maintain energy homeostasis through a balanced coupling of glycolysis and oxidative phosphorylation (OXPHOS). In osteoarthritis, mechanical stress and hypoxia stabilize HIF-1α, which upregulates key glycolytic enzymes (HK2, PKM2, LDHA) and enhances PDK-mediated inhibition of PDC, resulting in pathological glycolytic hyperactivation and impaired OXPHOS. This dual-engine imbalance leads to lactate accumulation, insufficient ATP production, and elevated ROS, thereby driving inflammatory responses, extracellular matrix (ECM) degradation, and chondrocyte dysfunction.Created with BioRender.com.

In healthy articular cartilage, chondrocytes—the sole resident cell type—exhibit a unique “dual-engine” metabolic profile. Evidence from human articular cartilage and ex vivo chondrocyte studies indicates that chondrocytes rely predominantly on glycolysis for ATP production even under normoxic conditions, while maintaining low but tightly regulated oxidative phosphorylation (OXPHOS) (21, 22). This glycolytic preference represents an adaptive strategy for survival within the intrinsically hypoxic cartilage microenvironment and provides biosynthetic intermediates required for extracellular matrix (ECM) synthesis. However, in osteoarthritis (OA), particularly knee OA, this finely tuned metabolic equilibrium collapses, manifesting as impaired OXPHOS concomitant with aberrant glycolytic upregulation, thereby establishing a metabolic foundation for chondrocyte dysfunction. These coordinated alterations in glycolysis and mitochondrial metabolism are schematically summarized in **Figure 1**.

During OA progression, preclinical studies in surgically induced knee OA models, especially the destabilization of the medial meniscus (DMM) mouse model, demonstrate that severe mitochondrial dysfunction renders OXPHOS—normally the high-efficiency metabolic “engine”—progressively inoperative. The pyruvate dehydrogenase complex (PDC), which links glycolysis to mitochondrial respiration, is negatively regulated by pyruvate dehydrogenase kinases (PDKs). In DMM-induced knee OA mice, downregulation of PDK1 in articular cartilage decreases phosphorylation of pyruvate dehydrogenase (PDH), thereby enhancing pyruvate conversion to acetyl-CoA and promoting tricarboxylic acid (TCA) cycle flux (23). Conversely, genetic PDK2 deficiency in mice augments OXPHOS activity and significantly attenuates cartilage degeneration in surgically induced knee OA, highlighting the pivotal regulatory role of PDK isoforms in OA-associated metabolic remodeling (24). In addition, both *in vitro* chondrocyte experiments and animal OA models implicate pyruvate kinase M2 (PKM2) in this metabolic imbalance. Recent mechanistic studies demonstrate that SIRT3-mediated deacetylation activates PINK1, which subsequently modulates PKM2 activity, driving a metabolic shift from glycolysis toward OXPHOS and improving mitochondrial ATP production (25, 26). Collectively, these findings—largely derived from preclinical models—suggest that OXPHOS collapse leads to ATP insufficiency, which is inadequate to sustain the high energetic demands of ECM synthesis and cartilage homeostasis, thereby impairing tissue repair and chondrocyte function (22).

Simultaneously, glycolysis becomes pathologically hyperactivated in OA, but this acceleration contributes more to inflammatory amplification than to effective energy compensation. Experimental evidence from *in vitro* chondrocyte cultures, animal OA models, and analyses of human OA cartilage indicates that inflammatory and hypoxic microenvironments stabilize hypoxia-inducible factor-1α (HIF-1α), a master regulator of metabolic reprogramming. HIF-1α upregulates key glycolytic enzymes, including hexokinase 2 (HK2), lactate dehydrogenase A (LDHA), and PDK1, thereby enhancing glycolytic flux while further suppressing mitochondrial respiration, forming a self-reinforcing metabolic loop (27). Lactate accumulation, primarily generated by LDHA, has been shown in rodent OA models and *in vitro* immune–chondrocyte interaction systems to directly promote joint inflammation and nociception (28), sustain pro-inflammatory M1 macrophage polarization, and exacerbate synovial inflammation (29–31). This emerging immunometabolic crosstalk appears to be a critical driver of OA progression. Although enhanced glycolysis supplies biosynthetic intermediates, transcriptomic and metabolic analyses of human OA cartilage and preclinical models suggest that these metabolites are preferentially diverted toward pathological processes—such as chondrocyte dedifferentiation and hypertrophic transformation—rather than toward normal ECM synthesis, thereby accelerating cartilage degeneration (32, 33).

Taken together, metabolic remodeling in osteoarthritic chondrocytes reflects a coupled disturbance characterized by impaired oxidative phosphorylation (OXPHOS) alongside dysregulated glycolysis, rather than a simple metabolic shift (21, 22). Evidence primarily from preclinical knee OA models, supported by observations in human cartilage, suggests that the PDK/HIF-1α axis contributes to this imbalance by linking mitochondrial dysfunction, energy deficiency, and altered glycolytic flux (23, 24, 27). As a consequence, increased reactive oxygen species (ROS) generation and lactate accumulation may facilitate activation of inflammatory pathways, including NLRP3 inflammasome signaling, thereby promoting a pro-inflammatory and catabolic microenvironment associated with extracellular matrix (ECM) degradation (28–31). Accordingly, modulation of this metabolic axis, such as targeting PDKs, LDHA, or HIF-1α, warrants further investigation as a potential disease-modifying approach in OA, particularly across different joint types and clinical contexts (32, 33).

### 2.2 Imbalance between ROS generation and antioxidant defense

Mitochondrial dysfunction in osteoarthritis (OA), predominantly characterized in articular chondrocytes from knee OA, is closely associated with redox imbalance, in which excessive reactive oxygen species (ROS) production overwhelms endogenous antioxidant defenses and contributes to disease progression. Unless otherwise specified, the mechanisms discussed in this section are primarily supported by evidence from chondrocyte-based *in vitro* studies, surgically induced OA animal models, and analyses of human OA cartilage specimens.

Key mitochondrial ROS species implicated in OA include superoxide anion (O_2_•^−^), hydrogen peroxide (H_2_O_2_), and hydroxyl radicals (•OH). Experimental studies using *in vitro* chondrocyte models and *in vivo* OA models have shown that dysfunction of the mitochondrial electron transport chain (ETC), triggered by OA-related stimuli such as mechanical overload and pro-inflammatory cytokines (e.g., IL-1β), enhances electron leakage and promotes excessive O_2_•^−^ generation (34, 35). Elevated mitochondrial ROS have been demonstrated in human OA chondrocytes to damage mitochondrial DNA (mtDNA) and impair mtDNA repair capacity (36). In parallel, ROS-driven lipid peroxidation leads to the accumulation of cytotoxic aldehydes, including malondialdehyde (MDA) and 4-hydroxynonenal (4-HNE), which have been linked to ferroptosis and apoptotic cell death in chondrocytes (37, 38). Notably, recent *in vitro* evidence indicates that sterol carrier protein 2 (SCP2) facilitates the transport of lipid hydroperoxides into mitochondria, thereby promoting mitochondrial-dependent ferroptosis in OA chondrocytes (38). Excessive ROS also induce mitochondrial permeability transition pore (mPTP) opening and Bax-mediated cytochrome c release, activating caspase cascades and apoptosis, as demonstrated in both OA cartilage samples and experimental models (39, 40).

**Figure 2:**
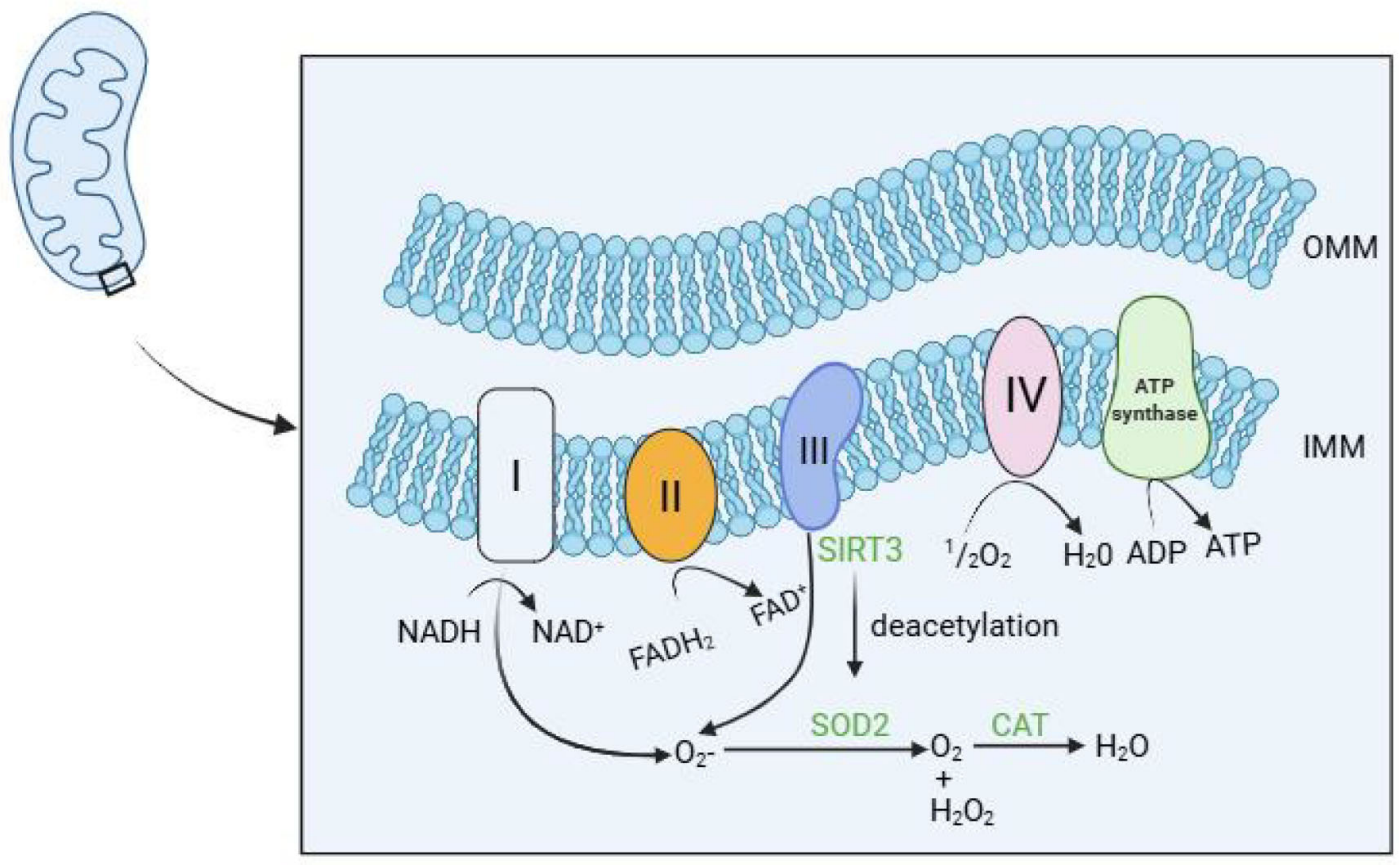
SIRT3-centered mitochondrial antioxidant circuitry in chondrocytes and its collapse during osteoarthritis.

To counteract oxidative stress, chondrocytes rely on multilayered antioxidant defense systems. Mitochondria-localized superoxide dismutase 2 (SOD2) constitutes the first line of defense against O_2_•^−^ and is tightly regulated by Sirtuin 3 (SIRT3)–mediated deacetylation. The hydrogen peroxide (H_2_O_2_) generated by SOD2 is subsequently detoxified into water and oxygen by catalase (CAT) and other peroxidases, thereby preventing secondary oxidative damage within the mitochondrial redox network. This coordinated mitochondrial redox network, involving ETC-derived ROS production, SIRT3-dependent SOD2 activation, and downstream H_2_O_2_ detoxification, is schematically illustrated in **Figure 2**. In OA, reduced SIRT3 expression or activity in chondrocytes has been shown to impair SOD2 function (41, 42). Mechanistically, SIRT3-mediated deacetylation is required for SOD2 activation, as demonstrated in non-OA cellular systems (43). In addition, SIRT3 activates isocitrate dehydrogenase 2 (IDH2) through deacetylation, thereby sustaining mitochondrial NADPH production and glutathione-dependent redox homeostasis (44). Interventions that restore SIRT3 activity, including caloric restriction or NAD^+^ precursor supplementation, have been shown in preclinical models to enhance SOD2 function and attenuate OA progression (45, 46).

In healthy cartilage, the mitochondrial deacetylase SIRT3 maintains redox homeostasis through two complementary mechanisms: direct deacetylation and activation of manganese superoxide dismutase (SOD2), the principal scavenger of mitochondrial superoxide (O_2_•^−^), and deacetylation of isocitrate dehydrogenase 2 (IDH2), thereby sustaining NADPH supply required for glutathione (GSH) recycling and thiol-based antioxidant systems. Consequently, reactive oxygen species (ROS) generated at the inner mitochondrial membrane (IMM) and within the matrix are efficiently neutralized, preserving chondrocyte viability and extracellular matrix (ECM) integrity. In osteoarthritis (OA), SIRT3 expression and activity are markedly downregulated, leading to hyperacetylation and enzymatic inactivation of SOD2, accumulation of O_2_•^−^, and diminished NADPH-dependent antioxidant capacity. The resulting ROS surge propagates mitochondrial dysfunction, chondrocyte senescence, and catabolic signaling that accelerate cartilage degradation. Pharmacological restoration of SIRT3 activity via caloric restriction mimetics or NAD^+^ precursors (e.g., NMN or NR) re-establishes SOD2- and IDH2-mediated antioxidant defenses, attenuates oxidative damage, and mitigates OA progression in preclinical models. OMM, outer mitochondrial membrane; IMM, inner mitochondrial membrane.Created with BioRender.com.

Beyond the SIRT3–SOD2 axis, nuclear factor erythroid 2–related factor 2 (NRF2)–mediated transcriptional programs play a central role in coordinating antioxidant responses. Under basal conditions, NRF2 is sequestered by Kelch-like ECH-associated protein 1 (Keap1) and targeted for ubiquitin-dependent degradation. Mitochondrial ROS oxidize cysteine residues on Keap1, allowing NRF2 to translocate into the nucleus, bind antioxidant response elements (AREs), and induce the transcription of phase II detoxifying and antioxidant genes, including heme oxygenase-1 (HO-1) and NAD (P)H quinone dehydrogenase 1 (NQO1) (47, 48). Studies in chondrocyte cultures and OA animal models demonstrate that activation of the NRF2/HO-1 axis reduces oxidative damage and apoptosis, thereby exerting chondroprotective effects (49–51).

mportantly, NRF2 signaling also regulates mitochondrial quality control beyond classical antioxidation. Preclinical studies indicate that NRF2 enhances mitophagy through PINK1 upregulation (52), suppresses excessive mitochondrial fission by promoting Drp1 degradation (53), and stimulates mitochondrial biogenesis via induction of nuclear respiratory factor 1 (NRF-1) (54). Through these mechanisms, NRF2 signaling contributes to the maintenance of mitochondrial integrity and metabolic homeostasis in OA chondrocytes. In parallel, excessive mitochondrial ROS amplify inflammatory signaling by promoting the release of damage-associated molecular patterns (DAMPs), such as mtDNA and cardiolipin, which activate the NLRP3 inflammasome and drive caspase-1–dependent maturation of IL-1β and IL-18. This process has been implicated in both synovial inflammation and cartilage degradation in experimental models of sterile arthritis and OA (55). Pharmacological or genetic activation of NRF2 has been shown to suppress NLRP3 inflammasome activation, whereas NRF2 inhibition exacerbates oxidative stress–driven inflammatory responses (56).

Collectively, current evidence—largely derived from cartilage-focused experimental systems—indicates that an imbalance between excessive mitochondrial ROS generation and impaired SIRT3–SOD2/NRF2-ARE antioxidant defenses represents a central pathogenic mechanism in OA. Although chondrocytes constitute the primary experimental focus, emerging data suggest that similar redox-driven mitochondrial dysfunction may also occur in other joint tissues, including synovium and subchondral bone, thereby contributing to the inflammatory and degenerative joint microenvironment. NRF2 agonists, such as tangeretin, and mitochondrial-targeted antioxidants, including Mitoquinone, have demonstrated chondroprotective and antioxidative effects *in vitro* and in preclinical OA models through enhancement of NRF2-dependent redox signaling and mitochondrial quality control (47, 49, 52). However, their therapeutic efficacy in OA patients has not yet been validated in clinical studies.

### 2.3 Imbalanced mitochondrial dynamics: a central driver of OA pathology

Mitochondrial dynamics—comprising the continuous balance of fission and fusion—are essential for maintaining chondrocyte homeostasis in knee osteoarthritis (OA). In the pathological microenvironment of OA cartilage, inflammatory mediators and abnormal mechanical loading disrupt this balance, leading to excessive mitochondrial fission and impaired fusion.

At the molecular level, evidence from *in vitro* studies using human and rodent knee OA chondrocytes demonstrates that pro-inflammatory cytokines (e.g., IL-1β, TNF-α) and mechanical stress activate signaling pathways that promote Dynamin-related protein 1 (Drp1) phosphorylation, a critical determinant of mitochondrial fission. extracellular signal–regulated kinase 1/2 (ERK1/2) -mediated phosphorylation of Drp1 at Ser616 enhances its GTPase activity and mitochondrial recruitment, thereby inducing mitochondrial fragmentation and apoptosis in chondrocytes (57, 58). In parallel, TANK-binding kinase 1 (TBK1)-dependent Drp1 Ser616 phosphorylation has been shown to regulate mitochondrial dynamics and autophagy activation in OA chondrocytes, primarily based on preclinical OA models (59).

TRIB3 upregulation further destabilizes the A-kinase anchoring protein 1 (AKAP1)/protein kinase A (PKA) complex, reducing inhibitory Ser637 phosphorylation of Drp1 and relieving its suppression, a mechanism originally characterized in degenerative cartilage-related tissues and subsequently supported in OA-relevant models (59).Additional regulators, including AMP-activated protein kinase (AMPK) and nuclear receptor subfamily 4 group A member 1 (NR4A1), modulate Drp1 activation under inflammatory stress in cellular and animal models of knee OA (60).

Conversely, mitochondrial fusion is markedly impaired during OA progression. optic atrophy 1 (OPA1), a key mediator of inner mitochondrial membrane fusion and cristae maintenance, is indispensable for cartilage integrity. Although the causal role of OPA1 loss has been most comprehensively demonstrated in murine models of intervertebral disc degeneration, accumulating evidence suggests that similar mitochondrial fusion defects operate in knee OA, given the shared biomechanical stress and chondrocyte-like cell phenotype (61). Mitofusin 2 (MFN2), which regulates outer membrane fusion and ER–mitochondria tethering, is significantly downregulated in knee OA chondrocytes derived from both animal models and human cartilage, correlating with metabolic dysfunction and inflammatory activation (62). Importantly, FGF18—evaluated in experimental knee OA models—restores mitochondrial dynamic balance by upregulating MFN2 and suppressing Drp1 activity via phosphoinositide 3-kinase (PI3K)–protein kinase B (AKT) signaling, thereby exerting chondroprotective effects and highlighting the pathogenic relevance of fusion defects in knee OA (63). Impaired fusion proteins diminish mitochondrial self-repair capacity, preventing compensation for localized damage and ultimately leading to collapse of the mitochondrial network.

**Figure 3:**
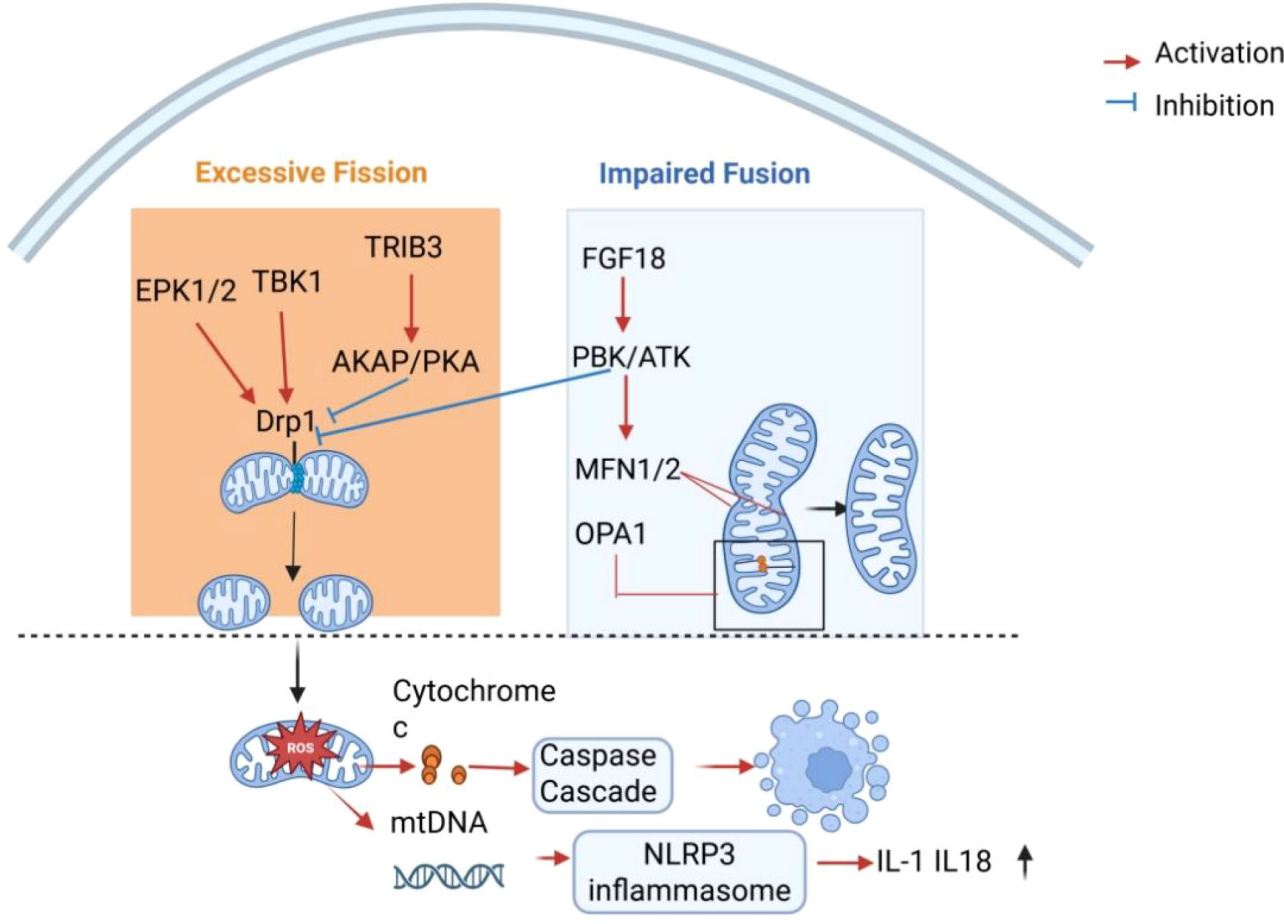
Imbalance of mitochondrial dynamics in osteoarthritis.

The imbalance in mitochondrial dynamics triggers downstream pathological cascades. As depicted in **Figure 3**, excessive Drp1-driven mitochondrial fission not only promotes cytochrome c–dependent apoptotic cascades but also facilitates mtDNA release and NOD-like receptor family pyrin domain containing 3 (NLRP3) inflammasome activation, thereby coupling mitochondrial structural disruption to inflammatory amplification in osteoarthritis. Fragmented mitochondria exhibit increased susceptibility to mitochondrial membrane potential (ΔΨm) collapse, cytochrome c release, and caspase activation, directly inducing chondrocyte apoptosis in *in vitro* and *in vivo* knee OA models (57, 64). Accumulation of dysfunctional mitochondria in chondrocytes constitutes a sustained source of excessive reactive oxygen species (ROS), leading to oxidative stress and procatabolic reprogramming that accelerates cartilage degeneration (64, 65). In parallel, evidence from synovial tissue indicates that mitochondrial DNA (mtDNA) leakage can activate the NLRP3 inflammasome, thereby amplifying inflammatory cascades within the joint microenvironment (66, 67). Together, these findings highlight that mitochondrial dysfunction contributes to osteoarthritis progression through cell-type–specific mechanisms in both cartilage and synovium.

Therapeutic strategies aimed at restoring mitochondrial dynamic homeostasis focus on both inhibiting pathological fission and enhancing fusion. Drp1 inhibition (e.g., Mdivi-1) or interventions promoting Ser637 phosphorylation via AKAP1/PKA signaling attenuate excessive fission in preclinical models of knee OA and related cartilage degeneration (64, 68). Enhancing MFN2 or OPA1 expression restores mitochondrial network integrity, with FGF18 providing proof-of-concept in knee OA models (63). Emerging nanotechnologies, such as chondrocyte-targeted exosome-mediated delivery of Nrf2, further demonstrate the translational potential of modulating mitochondrial dynamics in degenerative joint diseases (69).

Finally, Mitochondrial transplantation has recently been proposed as a novel mechanism of intercellular metabolic rescue in osteoarthritis. In human OA chondrocytes, Angela et al. demonstrated that functional mitochondria can be transferred via tunneling nanotubes (TNTs), leading to restoration of mitochondrial membrane potential, reduction of oxidative stress, and normalization of ATP production, thereby preserving cartilage matrix integrity (20). In parallel, Kwon et al. showed that mitochondrial transfer in an LPS-induced *in vitro* synovitis model restored fibroblast-like synoviocyte (FLS) plasticity and metabolic flexibility, supporting a broader role of intercellular mitochondrial exchange in joint inflammatory microenvironments (70).

Inflammatory cytokines and cellular stress signals promote dynamin-related protein 1 (Drp1)-mediated mitochondrial fission while suppressing optic atrophy 1 (OPA1)- and mitofusin 2 (MFN2)-dependent mitochondrial fusion, resulting in excessive mitochondrial fragmentation. This structural disruption leads to mitochondrial dysfunction characterized by cytochrome c release, enhanced oxidative stress, and mitochondrial DNA (mtDNA)-mediated activation of the NLRP3 inflammasome. Consequently, these events promote chondrocyte apoptosis, metabolic reprogramming, and inflammatory amplification during osteoarthritis progression.Created with BioRender.com.

### 2.4 Bidirectional regulation of mitophagy in osteoarthritis

**Figure 4:**
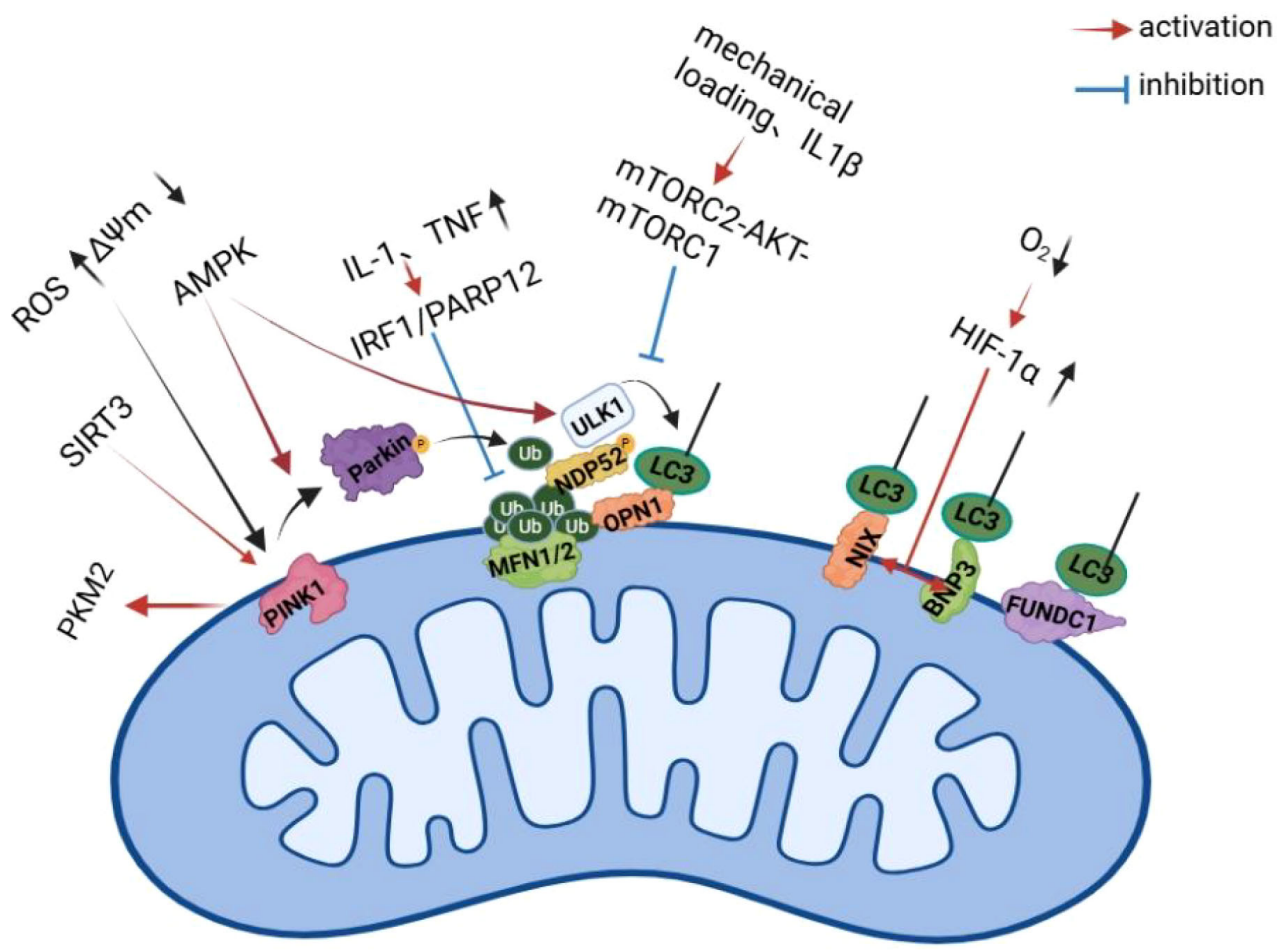
Regulatory networks of mitophagy in osteoarthritis chondrocytes.

During OA progression, mitophagy serves as a pivotal mitochondrial quality control mechanism, yet its regulation is highly context dependent and varies across disease stage, joint compartment, and experimental model. Most mechanistic insights to date have been derived from knee OA–related chondrocyte studies, including *in vitro* stimulation models and murine destabilization of the medial meniscus (DMM) models, with limited direct validation in human cartilage tissues. These regulatory nodes collectively converge on mitochondrial quality control pathways, linking inflammatory and metabolic stress to impaired mitophagy in osteoarthritic chondrocytes (**Figure 4**).

Inflammatory stressors (e.g., IL-1β, TNF-α) and mechanical injury disrupt mitophagy through multiple signaling nodes, among which dysfunction of the PTEN-induced putative kinase 1 (PINK1)–Parkin RBR E3 ubiquitin protein ligase (Parkin)pathway is particularly prominent. Under physiological conditions, PINK1 accumulates on depolarized mitochondria and recruits Parkin, which ubiquitinates outer mitochondrial membrane proteins such as mitofusin 1 and 2 (MFN1/2), enabling their recognition by autophagy receptors and subsequent clearance. In mouse knee OA models and primary chondrocytes, inflammatory stimulation has been shown to upregulate interferon regulatory factor 1 (IRF1), which transcriptionally induces poly (ADP-ribose) polymerase 12 (PARP12). PARP12 mediates poly (ADP-ribose) polymerase 12 (PARP12) and interferon-stimulated gene 15 (ISG15)–mediated conjugation (ISGylation) of MFN1/2, thereby impairing their ubiquitination, destabilizing PINK1 accumulation, and preventing Parkin recruitment. This mechanism suppresses mitophagy initiation and promotes the accumulation of dysfunctional mitochondria, contributing to cartilage degeneration *in vivo* (71).

Mitophagy is further suppressed through dysregulation of the UNC-51–like kinase 1 (ULK1) autophagy initiation complex. Under physiological conditions, ULK1 activity is dynamically controlled by the AMP-activated protein kinase (AMPK)–mechanistic target of rapamycin (mTOR) axis. However, in knee OA chondrocytes subjected to mechanical overload or IL-1β stimulation, rapamycin-insensitive companion of mTOR (RICTOR) expression is increased, enhancing mTORC2–AKT signaling and indirectly activating mTORC1. This locks ULK1 in an inactive state, impairs autophagosome formation, and leads to mitochondrial accumulation, excessive ROS production, and upregulation of catabolic enzymes such as MMP13 and. a disintegrin and metalloproteinase with thrombospondin motifs 5 (ADAMTS5). Importantly, genetic knockdown of RICTOR or pharmacological inhibition of protein kinase B (AKT) (MK-2206) in DMM mouse models restores autophagic flux and alleviates cartilage destruction, highlighting this pathway as a preclinical, but not yet clinically validated, regulator of mitophagy in OA (72).

In parallel with the canonical PINK1–Parkin pathway, hypoxia-driven receptor-mediated mitophagy pathways, including BNIP3/NIX- and FUN14 domain-containing protein 1 (FUNDC1)-dependent mechanisms, are activated in OA and summarized in **Figure 4**. Accumulated dysfunctional mitochondria act as persistent sources of ROS and release mitochondrial danger-associated molecular patterns, including mtDNA, which activate the NLRP3 inflammasome. This mechanism has been demonstrated not only in chondrocytes, but also in synovial macrophages, where impaired mitophagy promotes caspase-1 activation and pyroptotic cell death, thereby amplifying synovial inflammation and accelerating joint degeneration in experimental OA models (73, 74).

Beyond the canonical PINK1–Parkin pathway, receptor-mediated mitophagy contributes to OA pathogenesis in a hypoxia- and tissue-specific manner. Hypoxic conditions characteristic of osteoarthritic cartilage stabilize HIF-1α, which transcriptionally upregulates mitophagy receptors such as BNIP3 and NIX. Activation of this pathway has been demonstrated in human OA cartilage samples and murine knee OA models, where pharmacological stabilization of HIF-1α using dimethyloxalylglycine (DMOG) enhanced mitophagy and attenuated cartilage degeneration (75, 76). Similarly, dephosphorylated FUNDC1 activates mitophagy through its microtubule-associated protein 1 light chain 3 (LC3)-interacting region, with KD025 shown to ameliorate cartilage degeneration in preclinical OA models (77).

Upstream metabolic regulators further integrate mitophagy with cellular energy homeostasis. The sirtuin 3 (SIRT3)–PINK1–pyruvate kinase M2 (PKM2)axis, identified primarily in chondrocyte-based and animal studies, links mitochondrial deacetylation, mitophagy activation, and metabolic reprogramming toward efficient OXPHOS, thereby enhancing ATP production and redox balance (25, 26). AMPK acts as a central energy sensor across joint tissues, phosphorylating ULK1 and synergizing with PINK1–Parkin signaling. Natural compounds such as ginsenoside Rh1 and curcumin have been shown to enhance mitophagic flux and reduce oxidative stress in chondrocyte cultures and OA animal models, although clinical validation remains lacking (78, 79).

Importantly, mitophagy exerts a dual and stage-dependent role in OA. Insufficient mitophagy leads to toxic mitochondrial accumulation, whereas excessive activation may compromise cellular energy reserves and promote cell death. Consequently, emerging therapeutic strategies aim to restore mitophagy homeostasis rather than indiscriminately enhance it. These approaches—including PINK1–Parkin–activating compounds (e.g., acetyl zingerone), pH-responsive nanoparticles delivering ursolic acid or cerium dioxide, and adipose-derived mesenchymal stem cell exosomes—have demonstrated efficacy primarily in knee OA animal models and *in vitro* systems, and should currently be regarded as preclinical proof-of-concept interventions (80–82).

Collectively, these findings underscore mitophagy as a context-dependent, tissue-interactive regulator of OA progression, involving not only chondrocytes but also synovial macrophages and mesenchymal stromal cells. Future studies integrating human joint tissues and longitudinal clinical data are required to determine the translational relevance of mitophagy-targeted therapies across different OA phenotypes.

Inflammatory cytokines (IL-1β, TNF-α) activate IRF1/PARP12, disrupting MFN1/2 ubiquitination and impairing the PINK1–Parkin pathway. Mechanical loading and IL-1β trigger the mTORC2–AKT–mTORC1 axis, which inhibits ULK1-mediated autophagy initiation. Under hypoxia, HIF-1α stabilization upregulates BNIP3, NIX, and FUNDC1, which directly interact with LC3 to promote receptor-mediated mitophagy. Upstream regulators such as SIRT3 and AMPK enhance PINK1/Parkin-dependent mitophagy, whereas their dysfunction contributes to the accumulation of damaged mitochondria and oxidative stress in osteoarthritis progression.Created with BioRender.com.

## 3 Mitochondrial transplantation in the treatment of osteoarthritis

Following the detailed exploration of mitochondrial dysfunction in osteoarthritis (OA), it has become clear that the resulting energy crisis, oxidative stress, and programmed cell death represent progressive and self-reinforcing pathological drivers. Conventional therapeutic strategies for osteoarthritis have primarily aimed to indirectly modulate mitochondrial dysfunction at the preclinical level, mainly through pharmacological approaches such as enhancing endogenous antioxidant defenses, attenuating mitochondrial oxidative stress, or inhibiting excessive mitochondrial fission. Preclinical studies have shown that agents targeting mitochondrial ROS production, mitochondrial dynamics (e.g., DRP1-mediated fission), or redox homeostasis can partially alleviate chondrocyte catabolic responses and cartilage degeneration in cellular and animal models of OA (52, 57, 83, 84). However, these strategies remain largely experimental and have not yet translated into established clinical therapies for OA, owing to limited efficacy, poor tissue specificity, and potential off-target effects. Consequently, there is a growing recognition that indirect pharmacological modulation alone may be insufficient to reverse advanced mitochondrial dysfunction in osteoarthritic cartilage.In recent years, mitochondrial transplantation—a more direct and fundamental therapeutic paradigm—has gained prominence. This strategy seeks to deliver intact, functional mitochondria into damaged chondrocytes via endogenous or exogenous pathways, thereby “replacing” or “augmenting” their bioenergetic machinery and offering a novel perspective for restoring cellular homeostasis. In the following sections, we systematically discuss the therapeutic potential and latest advances of mitochondrial transplantation in OA, focusing on cell-based therapy, cell-free interventions, and engineered mitochondrial technologies. In the following sections, we systematically discuss the therapeutic potential and latest advances of mitochondrial transplantation in OA, focusing on cell-based therapy, cell-free interventions, and engineered mitochondrial technologies. A comparative overview of representative mitochondrial transplantation strategies, including donor cell sources, delivery routes, experimental models, and therapeutic outcomes, is summarized in **Table 1**.

**Table 1:** Preclinical and experimental evidence of mitochondrial transfer/transplantation in osteoarthritis.

Ref.	Transfer type	Experimental method	Model	Mechanism/Key findings
Wang et al., 2020, Chin Med J (95)	Cell-based (MSCs → Chondrocytes)	Co-culture of bone-marrow MSCs with rat chondrocytes; mitochondrial transfer tracked by MitoTracker	Rat chondrocytes *in vitro*	Transferred mitochondria improved mitochondrial function, rescued chondrocyte degeneration
Korpershoek et al., 2022, Cartilage (96)	Cell-based (MSCs → Chondrocytes)	MSC-chondrocyte 3D co-culture; mitochondrial DNA/proteoglycan deposition measured	Human 3D cartilage culture	Mitochondrial transfer increased mtDNA content and enhanced ECM proteoglycan deposition
Junyi et al., 2025, ACS Nano (97)	Cell-based (M-Sec overexpressed stem cells)	Genetic engineering to overexpress M-Sec; quantification of tunneling nanotubes (TNTs)	*In vitro*, multiple stem cell models	M-Sec promoted abundant TNT formation, enabling active mitochondrial transfer across cells
Matthew et al., 2022, Front Bioeng Biotechnol (112)	EV-based (MSC-derived EVs)	Isolation of extracellular vesicles; functional mitochondria detected within EVs	Human MSC culture	MSC-derived EVs carry intact mitochondria, capable of functional transfer to recipient cells
Xingfu et al., 2025, Int Immunopharmacol (113)	EV-based (Synovial MSC-derived EVs)	Collection of EVs from synovial fluid-derived MSCs; chondrocytes treated under stress/inflammation	Human OA chondrocytes *in vitro*; OA animal model	Mitochondria-rich EVs inhibited chondrocyte senescence and delayed OA progression
Ziyi et al., 2025, J Nanobiotechnology (114)	EV-based (Adipose MSC-derived EVs)	Adipose-derived EVs transferred into dendritic cells; mitochondrial tracking	Rat TMJOA model	EVs reprogrammed dendritic cells via mitochondrial transfer, alleviating TMJOA inflammation
Carlos et al., 2025, Cells (121)	Direct mitochondrial transplantation	Isolation of rat skeletal muscle mitochondria (differential centrifugation + OptiPrep); intra-articular injection; NIRF tracking	Rat OA model (surgical induction)	Transplanted mitochondria localized in cartilage >7 days, doubled ATP, reduced cytokines, improved OARSI score by 45%, with no systemic toxicity
Angela et al., 2024, Theranostics (20)	Direct mitochondrial transplantation (MSCs-derived mitochondria)	Optimized mitochondrial isolation from MSCs; co-incubation with chondrocytes; 2D + 3D cartilage chip validation	OA chondrocytes + cartilage-on-chip model	Transferred mitochondria increased ATP (~2.4×), reduced ROS, restored redox balance, preserved ECM
Lee et al., 2023 (122)	Direct mitochondrial transplantation	Intra-articular injection of exogenous mitochondria into OA animal models	OA animal model	Mitochondrial transplantation significantly delayed OA progression, improved chondrocyte viability, and attenuated cartilage degeneration
G. Zhong et al., 2024, Bone (125)	Direct mitochondrial transplantation + Gene therapy (rAAV-IGF-I)	Autologous mitochondrial transplantation combined with rAAV-IGF-I delivery into human OA chondrocytes	Human OA chondrocytes *in vitro*	Promoted IGF-I expression (~8×), improved mitochondrial dynamics, enhanced ECM synthesis, suppressed TNF-α
Fahey et al., 2022, Sci Rep (92)	Cell-based (MSCs → Chondrocytes)	Co-culture of MSCs with stressed chondrocytes (Rotenone/Antimycin, hyperoxia, or mechanical injury); mitochondrial labelling; gap-junction inhibition (Cx43); confocal microscopy + flow cytometry	*In vitro* (equine/murine chondrocytes under mitochondrial, environmental, mechanical stress) + ex vivo injured cartilage explants	MSCs donate healthy mitochondria under stress conditions, improving chondrocyte mitochondrial respiration & ATP production; transfer enhanced by stress (chemical, environmental, mechanical); inhibited by gap-junction blockers (Cx43)

MSCs, Mesenchymal stromal cells; EVs, Extracellular vesicles; TNTs, Tunneling nanotubes; OA, Osteoarthritis; TMJOA, Temporomandibular joint osteoarthritis; I/R, Ischemia-reperfusion.

### 3.1 Cell-based therapy: MSC-mediated mitochondrial transfer in osteoarthritis

As undifferentiated progenitor cells at the apex of the cell lineage hierarchy, stem cells possess remarkable self-renewal and multipotent differentiation capabilities, enabling them to generate a wide array of tissues, organs, and specialized cell types. This positions them as a cornerstone in tissue engineering and regenerative medicine. In rat models of knee osteoarthritis, intra-articular injection of bone marrow–derived mesenchymal stem cells (MSCs) demonstrated that MSCs alleviate synovitis and cartilage damage by upregulating IL-10 and downregulating TNF-α and IL-6, thereby promoting M1-to-M2 macrophage polarization. This study was the first to establish *in vivo* the mechanistic link between SPIO-labeled MSC persistence and their anti-inflammatory and immunomodulatory functions (85). Clinically, two randomized, double-blind/triple-blind, placebo-controlled trials further confirmed the efficacy and safety of MSCs in knee OA therapy (86, 87). Nevertheless, the precise molecular and cellular mechanisms underlying their therapeutic effects remain incompletely elucidated.

It is now well recognized that the therapeutic effects of MSCs in osteoarthritis are multifactorial, involving immunomodulation, paracrine signaling, extracellular vesicle secretion, and regulation of inflammatory cell phenotypes. Beyond these established mechanisms, recent evidence indicates that MSCs can also repair respiratory dysfunction, initiate cellular reprogramming, and restore cell viability through mitochondrial (MT) transfer (88–90).

Owing to their glycolysis-dominant metabolic phenotype, low energy demand, and inherent tropism toward injured tissues, undifferentiated MSCs represent an efficient donor population for mitochondrial transfer (91). In addition, their immune-privileged properties and tightly regulated redox balance further support their role as a source of functionally intact mitochondria under pathological conditions. Importantly, mitochondrial transfer is markedly enhanced in stress contexts. Fahey et al. demonstrated that MSCs donate mitochondria to injured chondrocytes exposed to chemical, inflammatory, or mechanical stress, restoring mitochondrial membrane potential, reducing ROS levels, and suppressing apoptosis within 4–6 hours (92). Collectively, these findings suggest that mitochondrial transfer constitutes an emerging and complementary mechanism by which MSCs support joint homeostasis and cartilage repair, rather than acting as the sole mediator of their therapeutic efficacy in OA.

At the same time, injured cells release dysfunctional mitochondria and mtDNA as “danger signals” to stimulate MSC mitochondrial donation (93), while the pathological microenvironment facilitates the formation of transfer conduits such as TNTs (94).

The central role of mitochondrial transfer in MSC-mediated OA therapy is becoming increasingly evident. In 2019, co-culture experiments first confirmed that chondrocytes with mitochondrial dysfunction emit “distress signals, “ prompting MSCs to donate functional mitochondria via TNTs. In 2020, Wang et al. further demonstrated that BMSC-derived mitochondria integrate into the mitochondrial network of OA chondrocytes, directly restoring membrane potential and ATP production, suppressing catabolic gene expression (e.g., MMP-13), and maintaining chondrocyte phenotype. However, this study remained largely descriptive, leaving unanswered questions regarding the molecular switches and transfer conduits involved (95).

Korpershoek and colleagues classified three major transfer mechanisms: (i) direct cell–cell contact; (ii) tunneling nanotube (TNT) networks; and (iii) extracellular vesicles (EVs). They demonstrated that MSCs can transfer mitochondria into chondrocytes through these pathways, increasing mtDNA copy number, elevating ATP levels, reducing ROS within 4–6 hours, and promoting significant proteoglycan (particularly aggrecan) deposition in chondrocytes after 48 hours (96). Genetic engineering approaches that overexpress M-Sec, Miro1, or Connexin-43 in MSCs markedly enhanced TNT formation and facilitated efficient transfer of mitochondria, lysosomes, and regulatory miRNAs to damaged chondrocytes, macrophages, or neurons *in vitro* and in rodent OA models. This “overexpression–multi-TNT” strategy significantly restored mitochondrial membrane potential (1.8–2.3-fold increase), boosted ATP production (2.0–2.4-fold increase), reduced ROS and inflammatory mediators, and ultimately attenuated cartilage degeneration, osteophyte formation, and pain, establishing a new paradigm for TNT-mediated multi-component cell-free OA therapy (97).

Connexin 43 (Cx43) has been identified as an important regulator of intercellular mitochondrial transfer between mesenchymal stromal cells (MSCs) and chondrocytes. Recent evidence demonstrates that Cx43 participates in the formation of tunneling nanotube (TNT)-like intercellular connections in MSC–chondrocyte co-culture systems, thereby facilitating mitochondrial trafficking. In human MSC–chondrocyte co-culture models, Cx43 overexpression significantly increased the number of TNT-like structures and enhanced mitochondrial transfer efficiency, whereas CRISPR–Cas9–mediated Cx43 knockdown or pharmacological inhibition with carbenoxolone reduced mitochondrial transfer by more than 60%, concomitantly attenuating MSC-mediated chondroprotective effects (98). Beyond its role in intercellular mitochondrial exchange, Cx43 also plays a critical role in regulating chondrocyte plasticity and osteoarthritis pathophysiology. Elevated Cx43 expression has been observed in early-stage OA cartilage and correlates with Twist-1 upregulation, chondrocyte senescence, and extracellular matrix degradation. Conversely, targeted downregulation of Cx43 reverses chondrocyte dedifferentiation and restores cartilage matrix markers, including COL2A1 and proteoglycan deposition, underscoring its dual role in both disease progression and therapeutic responsiveness (99). Together, these findings suggest that Cx43 functions as a key molecular regulator linking MSC–chondrocyte mitochondrial transfer with chondrocyte fate decisions in OA, although its precise mechanistic integration across different disease stages warrants further investigation.

Despite their clinical promise, MSCs as “living drugs” face significant translational hurdles in orthopedics. Challenges include poor survival and engraftment in the inflamed, hypoxic intra-articular microenvironment, leading to transient therapeutic effects; pronounced heterogeneity in mitochondrial donation capacity among MSCs from different donors and batches, resulting in inconsistent clinical outcomes; and potential safety risks, including tumorigenicity and immune rejection, which remain a regulatory concern despite low incidence (100, 101). Furthermore, the high costs of culture, quality control, storage, and transportation of MSC-based products limit their accessibility as universal therapeutics.

#### Other cellular sources of mitochondria

Platelets have been investigated as an alternative source of mitochondria due to their abundance and ease of isolation. *In vitro* studies have demonstrated mitochondrial transfer from human platelets to fibroblasts, where platelet-derived mitochondria were internalized and contributed to the restoration of mitochondrial function in recipient cells (102)and promoted wound closure by modulating ROS levels in dermal fibroblasts (103). Platelet-mediated mitochondrial transfer has also been shown to enhance adipose-derived stem cell viability and antioxidant capacity in preclinical models (104).

Moreover, methodological work using magnetically assisted mitochondrial transfer into cultured fibroblasts illustrates that mitochondria from donor cells can be delivered into fibroblast cytoplasm and increase cellular respiration, supporting the technical feasibility of fibroblast mitochondrial uptake in experimental systems (105). Although these alternative sources remain less extensively studied in osteoarthritis models, they broaden the conceptual framework of mitochondrial donation and warrant further investigation.

### 3.2 The rise of cell-free therapies

An emerging concept in cell-free therapy is to regard mitochondria themselves as “active biologics” or “organelle-based therapeutics, “ which can be produced ex vivo and directly delivered to diseased tissues to restore bioenergetic and redox homeostasis (18, 106, 107). Compared with cell-based approaches, this strategy aims to avoid inherent risks such as poor cell survival, uncontrolled differentiation, and immunogenicity. However, its translational feasibility critically depends on three key pillars: efficient isolation of highly functional mitochondria, effective *in vivo* delivery and tissue targeting strategies, and rigorous safety validation in both *in vitro* and *in vivo* systems.

#### Extracellular vesicle (EV)-mediated mitochondrial delivery

EVs are heterogeneous membrane-bound structures secreted by diverse cell types, carrying proteins, nucleic acids, and organelle components that play vital roles in intercellular communication and homeostasis (108). Recent studies have revealed that EVs not only transport conventional signaling molecules but can also encapsulate intact mitochondria, mitochondrial components, and free mtDNA, termed mitochondrial EVs (MitoEVs). For example, in ischemic stroke, MitoEVs delivered functional mitochondria to damaged brain endothelial cells, enhancing their survival (109). In acute lung injury models, adipose-derived MSC (AD-MSC)–derived EVs similarly transferred mitochondria to restore airway metabolic balance (110). These findings provide critical theoretical support for OA therapy.

*In vitro* and animal experiments consistently demonstrate that MSC-derived exosomes (from umbilical cord or bone marrow) can block the OA inflammation–pyroptosis–pain axis through multiple mechanisms (111), though comprehensive mechanistic understanding remains incomplete. In OA research, Thomas et al. first confirmed that human BMSCs not only transfer mitochondria via TNTs but also actively package intact mitochondria into small EVs (EV-mito). These EVs restored mitochondrial membrane potential and ATP production in recipient cells, establishing MSC-derived EVs as effective carriers for “cell-free mitochondrial transplantation” (112). Xingfu L. et al. isolated mt-enriched EVs (mt-EVs) from BMSCs and co-cultured them with chondrocytes, demonstrating that mt-EVs not only restored energy metabolism but also upregulated longevity-associated proteins such as SIRT3, counteracting cellular senescence. A single intra-articular injection provided long-lasting (≥14 days) chondroprotection without toxicity, representing a safer and more efficient cell-free strategy (113). In another study, Ziyi M. and colleagues showed that small EVs (sEVs) from human adipose-derived stem cells, upon intravenous administration, homed specifically to synovial dendritic cells (DCs) in OA joints. These DCs, “rejuvenated” by mitochondrial delivery, restored metabolic function (JC-1 ↑1.9-fold, ATP ↑2.1-fold), shifted from a pro-inflammatory M1 phenotype to an anti-inflammatory, tolerogenic M2 phenotype, and thereby ameliorated OA by modulating systemic immune microenvironments—highlighting the feasibility of non-invasive systemic therapy (114).

Notably, under stress or inhibitory conditions, MSC-derived MitoEVs may contain impaired mitochondria, whereas pretreatment with mitochondrial-protective peptides increases the loading of functional mitochondria (112). Although MitoEV-mediated transfer is generally less efficient than direct mitochondrial delivery—likely due to culture conditions such as incubation duration and vesicle dose—BMSC-derived microvesicles exhibited stronger efficacy in ameliorating IL-1β–induced mitochondrial dysfunction, underscoring the therapeutic promise of MitoEVs (115).

Despite these advances, several challenges remain. First, MitoEVs contain limited mitochondrial cargo, insufficient for large-scale therapeutic needs. Second, heterogeneity across cell sources and isolation techniques leads to significant compositional variability, impairing treatment consistency and reproducibility. While preliminary evidence suggests that donor cell pretreatment can increase functional mitochondrial loading and enhance therapeutic efficacy (116, 117), the efficient and stable isolation and enrichment of MitoEVs remains a bottleneck for clinical translation. Of note, recent findings indicate that adipocytes under energetic stress release sEVs carrying mitochondria, which migrate to the heart and enhance metabolic adaptability (118). Moreover, emerging techniques now allow selective isolation of specialized vesicles containing intact mitochondrial components, offering novel strategies to improve mitochondrial transfer efficiency (119).

### 3.3 Non-contact mitochondrial transfer in osteoarthritis

Although natural intercellular mitochondrial transfer has been demonstrated to improve cellular function in multiple disease models, its clinical translation is constrained by donor cell heterogeneity, low transplantation efficiency, and unstable efficacy. To address these limitations, researchers have developed non-contact mitochondrial transfer (NMT), whereby isolated mitochondria are delivered exogenously, circumventing risks such as immune rejection and tumorigenicity associated with cell-based therapy. Mechanistically, isolated mitochondria are actively internalized by recipient cells through macropinocytosis and endocytosis. Pioneering work by Tachibana et al. introduced healthy mitochondria into oocytes carrying mutant mtDNA, establishing the precedent for exogenous mitochondrial therapy (120). Li et al. found that under hypoxic co-culture conditions, motor neurons internalized exogenous mitochondria and improved ATP production (104). Masuzawa et al. further confirmed in a rabbit myocardial ischemia model that mitochondrial transplantation restored cardiac energy metabolism and alleviated reperfusion injury within hours (106). These studies laid the theoretical and technical foundation for NMT.

In OA, NMT research has expanded to evaluate feasibility, safety, mechanisms, and delivery optimization. Carlos et al. isolated mitochondria from rat skeletal muscle using differential centrifugation and OptiPrep density gradients, then injected them intra-articularly into OA models. Near-infrared fluorescence (NIRF) labeling and *in vivo* imaging demonstrated mitochondrial retention in cartilage for over 7 days, with uptake by chondrocytes. Functionally, a single injection doubled ATP levels in chondrocytes, reduced inflammatory cytokines in synovial fluid, and improved OARSI scores by ~45%, without evidence of immune rejection or systemic toxicity, providing critical evidence for the feasibility and safety of mitochondria as therapeutic agents (121). Similarly, Lee et al. reported that mitochondrial transplantation significantly delayed OA onset and progression, enhanced chondrocyte viability, and mitigated cartilage degeneration, further validating its *in vivo* therapeutic potential (122).

Building on the concept of non-contact mitochondrial transfer, Angel et al. further demonstrated that isolated mitochondria can function as bioactive organelles independent of their cellular origin. Using an optimized protocol to purify mitochondria from MSCs (as discussed in Section 2.1), the authors co-cultured these cell-free mitochondria with OA chondrocytes and observed efficient mitochondrial internalization. This uptake restored metabolic homeostasis, increased intracellular ATP levels by approximately 2.4-fold, reduced ROS accumulation, and preserved extracellular matrix integrity. Importantly, these protective effects were recapitulated in a three-dimensional biomimetic cartilage-on-chip model, underscoring the translational relevance of non-contact mitochondrial delivery beyond conventional two-dimensional culture systems (20). David et al. examined the clinical translational potential of NMT, comparing *in vitro* and *in vivo* models. They observed that while exogenous mitochondria rapidly entered cells and restored metabolism *in vitro*, they were more prone to degradation and clearance *in vivo*, attenuating efficacy. In a mouse acute hepatic ischemia–reperfusion model, intravenous injection of 100 μg mitochondria reduced serum ALT/AST, suggesting applicability across non-orthopedic conditions. This study emphasized the need for standardized protocols for mitochondrial preparation, dosing, and delivery to ensure reproducibility across disease models (123).

Emerging approaches such as MitoPunch, MitoCeption, and magnetic nanoparticle-assisted delivery have further improved efficiency and stability of mitochondrial transfer. Nonetheless, *in vivo* persistence of exogenous mitochondria remains limited, with some evidence suggesting degradation within one week (124). Future efforts must therefore focus on prolonging mitochondrial survival, enhancing delivery efficiency, minimizing invasiveness, and thoroughly assessing immunological safety and long-term efficacy.

Collectively, NMT in OA models has demonstrated robust potential in restoring energy metabolism, suppressing inflammation, and promoting cartilage repair. The successful application of allogeneic mitochondria and proposals for standardized strategies mark a pivotal step toward the clinical translation of “mitochondrial therapeutics.”

### 3.4 Engineered mitochondrial transplantation in osteoarthritis

Traditional naked mitochondrial transplantation is constrained by susceptibility to damage during isolation and storage, as well as inefficient uptake that largely relies on endocytosis. To overcome these barriers, research has shifted toward engineered mitochondrial transplantation (EMT), which can be broadly categorized into endogenous enhancement, exogenous modification, and physical facilitation.

#### 3.4.1 (1) Endogenous interventions: creating “super mitochondria.”

Genetic or pharmacological pretreatment of donor cells can optimize mitochondrial quality prior to isolation. For instance, Sun et al. activated ALDH2 in cardiomyocytes using Alda-1, producing mitochondria with improved survival and fusion capacity, which markedly enhanced repair in ischemia–reperfusion models (126). A more representative strategy involved engineering human umbilical cord MSCs to overexpress Miro1 (MitoHigh-MSC-mt), which exhibited superior intra-articular retention (≥21 days), robust restoration of energy metabolism (ATP ↑2.3-fold), and remodeling of antioxidant defenses, resulting in ~55% reduction in OARSI scores in OA models (20). Similarly, Zhong et al. developed an autologous mitochondria/rAAV-IGF-I platform, which increased IGF-I expression ~8-fold in OA chondrocytes. Embedded in PF127 hydrogel, this approach promoted cell proliferation, survival, ECM production, and balanced mitochondrial fusion/fission, while significantly reducing inflammatory mediators such as TNF-α, highlighting the therapeutic synergy of combined gene and mitochondrial therapy (125).

#### 3.4.2 (2) Exogenous modifications: building “Trojan horses.”

To address inefficient uptake of naked mitochondria, bioengineering approaches employ surface modifications or encapsulation. Triphenylphosphonium (TPP^+^) and its derivatives have been widely used for mitochondrial targeting, enhancing tissue-specific homing (127, 128). Dex-TPP modification increased mitochondrial internalization efficiency by approximately threefold (129, 130). In OA studies, fusogenic liposomes were used to encapsulate isolated mitochondria, generating biomimetic fusion mitochondria (FL-MT). By bypassing endocytosis, FL-MT achieved highly efficient delivery, with *in vitro* assays showing order-of-magnitude increases in uptake, ~2.3-fold elevation in ATP, and *in vivo* intra-articular injections prolonging mitochondrial retention to ≥21 days while significantly improving cartilage structure (131). This represents a “Trojan horse” delivery strategy integrating biomaterials with cell therapy.

#### 3.4.3 (3) Functional synergy: building composite therapeutic platforms.

Beyond energy supplementation, EMT can be combined with anti-inflammatory and epigenetic interventions. A recent study developed a gene-engineered biomimetic nanoplatform (HKL-GECM@MPNPs), encapsulating honokiol (HKL), a mitochondrial-targeting agent, within a cartilage cell membrane overexpressing IL-1R2. This design employed IL-1R2 as a “decoy” to neutralize IL-1β and improve the microenvironment, while delivering HKL directly to mitochondria to restore SIRT3 activity and reverse pathological phenotypes. In mouse OA models and human cartilage explants, this platform alleviated inflammation, reduced pain, and promoted cartilage repair (132). Such “inflammation neutralization + energy replenishment + epigenetic regulation” illustrates the synergistic potential of multidimensional interventions.

#### 3.4.4 (4) Physical facilitation: enhancing mitochondrial quality and uptake.

Beyond biochemical and genetic strategies, physical interventions have emerged as auxiliary approaches to enhance mitochondrial quality, bioenergetic performance, and intercellular transfer efficiency. Modalities such as photobiomodulation (PBM), ultrasound, and electroporation have been shown to directly modulate mitochondrial function and release.

PBM, particularly in the near-infrared range (e.g., 810 nm), enhances mitochondrial membrane potential and ATP synthesis by stimulating cytochrome c oxidase (COX), thereby improving oxidative phosphorylation efficiency and cellular stress resilience (133). This COX-dependent enhancement of ΔΨm and ATP availability provides a favorable bioenergetic state that may facilitate mitochondrial survival, uptake, and functional integration during engineered mitochondrial transplantation (EMT).

Importantly, accumulating preclinical evidence in osteoarthritis (OA) models demonstrates that PBM attenuates cartilage degeneration, suppresses inflammatory mediator production, and restores chondrocyte mitochondrial function, including improvements in ATP generation, redox homeostasis, and anabolic metabolism (134, 135). Consistently, systematic reviews and meta-analyses indicate that PBM alleviates pain and improves functional outcomes in patients with knee OA, supporting its translational relevance, albeit with moderate evidence certainty (136).

In addition, ultrasound-based physical stimulation has been reported to promote mitochondrial release from donor cells such as platelets, with the isolated mitochondria subsequently taken up and functionally utilized by recipient endothelial cells (104). Although direct evidence linking ultrasound-facilitated mitochondrial transfer to OA therapy remains limited, these findings provide a mechanistic rationale for its potential application in osteoarthritic tissues.

Collectively, physical facilitation strategies offer complementary means to optimize mitochondrial quality and delivery efficiency. When integrated with endogenous mitochondrial optimization and engineered delivery platforms, these modalities help overcome key limitations of naked mitochondrial transplantation. Preclinical studies in OA support the multidimensional benefits of EMT in restoring energy metabolism, remodeling the inflammatory microenvironment, and promoting cartilage repair, positioning EMT as a promising and multifunctional form of mitochondrial therapeutics.

## 4 Opportunities and challenges

### 4.1 Opportunities

Mitochondrial transplantation offers a paradigm-shifting opportunity for OA therapy. By directly targeting the core deficit of chondrocyte bioenergetics, it holds the potential to overcome the “symptom management without disease modification” bottleneck of current drugs, paving the way toward the first truly disease-modifying therapy. With advances in MSC culture, EV isolation, and gene editing, scalable platforms for high-quality, engineered mitochondria are becoming increasingly feasible. Concurrently, progress in targeted delivery, nanocarriers, and hydrogel-based sustained release offers versatile tools to overcome retention challenges in cartilage. Importantly, the large OA patient population and substantial unmet clinical needs create a favorable environment for regulatory prioritization, investment, and multicenter clinical trials. Once early clinical validation succeeds, mitochondrial transplantation could rapidly expand to other degenerative joint disorders, catalyzing growth in regenerative medicine markets.

### 4.2 Challenges

Several hurdles must be addressed before clinical implementation:

#### 4.2.1 Standardization bottlenecks

Lack of consensus on donor cell sources, mitochondrial extraction methods, and potency quality control leads to significant batch-to-batch variability, impeding GMP-compliant production.

#### 4.2.2 Delivery and retention issues

Rapid clearance from the joint cavity and limited cartilage penetration result in substantial mitochondrial loss within 24 hours; current carriers face challenges including low fusion efficiency, poor membrane integrity, and accelerated immune clearance.

#### 4.2.3 Safety concerns

The long-term persistence of allogeneic mtDNA raises unanswered questions about immune rejection, inflammatory memory, or tumorigenic risk; repeated dosing, metabolic overload, and ectopic distribution require rigorous toxicological evaluation.

#### 4.2.4 Mechanistic blind spots

How exogenous mitochondria integrate into host networks, whether they remodel epigenetics, and whether they suppress endogenous biogenesis remain largely unknown, restricting precision optimization.

#### 4.2.5 Translational gaps

Absence of standardized dose–response curves, stratification criteria, objective clinical endpoints, and long-term follow-up protocols impedes clinical trial design. Regulatory classification (cell therapy, biologic, or tissue-engineered product) remains unclear, adding uncertainty in approval timelines and costs.

#### 4.2.6 Regulatory considerations for mitochondrial transplantation

The clinical translation of mitochondrial transplantation faces a complex and evolving regulatory landscape. Unlike conventional small-molecule drugs, mitochondrial transfer strategies may be variably classified as cell-based therapies, biological products, or advanced therapy medicinal products, depending on the source of mitochondria, the method of delivery, and the degree of manipulation. Key regulatory challenges include standardization of mitochondrial isolation and quality control, assessment of immunogenicity and biodistribution, and long-term safety monitoring. At present, the absence of unified regulatory guidelines underscores the need for early dialogue between researchers and regulatory agencies to facilitate the responsible development of mitochondrial-based therapies.

## 5 Conclusion

Growing evidence supports the view that osteoarthritis is not merely a mechanically driven degenerative disorder but a chronic immunometabolic disease in which mitochondrial dysfunction integrates energy failure, oxidative stress, and innate immune activation. Impaired mitochondrial quality control in chondrocytes and synovial cells promotes mitochondrial DAMP–mediated inflammatory signaling, disrupts tissue homeostasis, and accelerates cartilage degeneration.

Within this framework, mitochondrial transplantation represents a paradigm shift from indirect modulation of inflammatory or metabolic pathways toward direct organelle-level restoration of cellular bioenergetics and immune balance. By replenishing functional mitochondria, this strategy has the potential to simultaneously restore oxidative phosphorylation, suppress mitochondrial-driven inflammatory amplification, and interrupt self-sustaining immunometabolic circuits that perpetuate joint damage.

Nevertheless, several challenges must be addressed before clinical translation, including standardization of mitochondrial quality, optimization of intra-articular delivery and retention, immunological safety, and regulatory classification. Future studies should focus on defining OA subtypes with heightened mitochondrial vulnerability, elucidating immune–mitochondrial crosstalk within the joint microenvironment, and developing precision-engineered delivery platforms. With rigorous preclinical validation and well-designed clinical trials, mitochondrial transplantation may emerge as a disease-modifying strategy for osteoarthritis and other chronic inflammatory joint diseases.
